# FoodKG: A Tool to Enrich Knowledge Graphs Using Machine Learning Techniques

**DOI:** 10.3389/fdata.2020.00012

**Published:** 2020-04-29

**Authors:** Mohamed Gharibi, Arun Zachariah, Praveen Rao

**Affiliations:** ^1^Department of Computer Science and Electrical Engineering, University of Missouri-Kansas City, Kansas City, MO, United States; ^2^Department of Electrical Engineering and Computer Science, University of Missouri-Columbia, Columbia, MO, United States; ^3^Department of Health Management and Informatics, University of Missouri-Columbia, Columbia, MO, United States

**Keywords:** machine learning, graph embeddings, knowledge graphs, AGROVOC, semantic similarity

## Abstract

While there exist a plethora of datasets on the Internet related to Food, Energy, and Water (FEW), there is a real lack of reliable methods and tools that can consume these resources. This hinders the development of novel decision-making applications utilizing knowledge graphs. In this paper, we introduce a novel software tool, called FoodKG, that enriches FEW knowledge graphs using advanced machine learning techniques. Our overarching goal is to improve decision-making and knowledge discovery as well as to provide improved search results for data scientists in the FEW domains. Given an input knowledge graph (constructed on raw FEW datasets), FoodKG enriches it with semantically related triples, relations, and images based on the original dataset terms and classes. FoodKG employs an existing graph embedding technique trained on a controlled vocabulary called AGROVOC, which is published by the Food and Agriculture Organization of the United Nations. AGROVOC includes terms and classes in the agriculture and food domains. As a result, FoodKG can enhance knowledge graphs with semantic similarity scores and relations between different classes, classify the existing entities, and allow FEW experts and researchers to use scientific terms for describing FEW concepts. The resulting model obtained after training on AGROVOC was evaluated against the state-of-the-art word embedding and knowledge graph embedding models that were trained on the same dataset. We observed that this model outperformed its competitors based on the Spearman Correlation Coefficient score.

## 1. Introduction

Food, energy, and water are the critical resources for sustaining human life on Earth. Currently, there are a plethora of datasets on the Internet related to FEW resources. However, there is still a lack of reliable tools that can consume these resources and provide decision-making capabilities (Rao et al., 2016). Moreover, FEW data exists on the Internet in different formats with different file extensions, such as CSV, XML, and JSON, and this makes it a challenge for users to join, query, and perform other tasks (Knoblock and Szekely, 2015). Generally, such data types are not consumable in the world of Linked Open Data (LOD), and neither are they ready to be processed by different deep learning networks (Meester, 2018). Recently, in September 2018, Google announced its “Google Dataset Search”, which is a search engine that includes graphs and Linked Data. Google Dataset Search is a giant leap in the Semantic Web domain, but the challenge is the lack of published knowledge graphs, especially in the FEW systems area (Gharibi et al., 2018).

Knowledge graphs, including Freebase (Bollacker et al., 2008), DBpedia, (Auer et al., 2007), and YAGO (Suchanek et al., 2007), have been commonly used in Semantic Web technologies, Linked Open Data, and cloud computing (Dubey et al., 2018) due to their semantic properties. In recent years, many free and commercial knowledge graphs have been constructed from semi-structured repositories like Wikipedia or harvested from the Web. In both cases, the results are large global knowledge graphs that have a trade-off between completeness and correctness (Hixon et al., 2015). Recently, different refinement methods have been proposed to utilize the knowledge in these graphs to make them more useful in domain-specific areas by adding the missing knowledge, identifying error pieces, and extracting useful information for users (Paulheim, 2017). Furthermore, knowledge extraction methods used in most of the knowledge graphs are based on binary facts (Ernst et al., 2018). These binary facts represent the relations between two entities, which limit their deep reasoning ability when there are multiple entities, especially in domain-specific areas like FEW (Vashishth et al., 2018).

The lack of reliable knowledge graphs serving FEW resources has motivated us to build our tool, FoodKG, which uses domain-specific graph embeddings to help in the decision-making process, improving knowledge discovery, simplifying access, and providing better search results. FoodKG enriches FEW datasets by adding additional knowledge and images based on the semantic similarities (Varelas et al., 2005) between entities within the same context. To achieve these tasks, FoodKG employs a recent graph embedding technique based on self-clustering called GEMSEC (Rozemberczki et al., 2019), which was retrained on the AGROVOC (Caracciolo et al., 2013) dataset. AGROVOC is a collection of vocabularies that covers all areas of interest to the Food and Agriculture Organization of the United Nations, including food, nutrition, agriculture, fisheries, forestry, and the environment. The retrained model, AGROVEC, is a domain-specific graph embedding model that enables FoodKG to enhance knowledge graphs with the semantic similarity scores between different terms and concepts. In addition, FoodKG also allows users to query knowledge graphs using SPARQL through a friendly user interface.

In this paper, we have proposed a tool called FoodKG that refines and enriches FEW resources to utilize the knowledge in FEW graphs in order to make them more useful for researchers, experts, and domain users. Our work makes several key contributions:

- FoodKG is a novel software tool that aims to enrich and enhance FEW graphs using multiple features. Adding a context to the provided triples is one of the first features that allows querying the graphs more easily and providing better input for deep learning models.
- FoodKG provides different Natural Language Processing (NLP) techniques, such as POS tagging, chunking, and Stanford Parser, to extract the meaningful subjects, unify the repeated concepts, and link related entities together (Klein and Manning, 2003; Chen and Manning, 2014; Manning et al., 2014).
- FoodKG employs the Specialization Tensor Model (STM) (Glavaš and Vulicć, 2018) to predict the newly added relations within the graph.
- We adopted WordNet (Miller, 1995) to return all the offsets for the provided subjects in order to parse the related images from ImageNet (Russakovsky et al., 2015). These images will be added to the graph in the form of Universal Resource Locator (URL) as related and pure images.
- FoodKG utilizes the GEMSEC (Rozemberczki et al., 2019) model that was retrained on AGROVOC with fine-tuning to produce AGROVEC to provide the semantic similarity scores between the similar and linked concepts. AGROVEC was compared with word embeddings and knowledge graph embedding models trained on the same dataset. By virtue of being trained on domain-specific graph data, AGROVEC achieved a superior performance to its competitors in terms of the Spearman Correlation Coefficient score.

Our results showed that AGROVEC provides more accurate and reliable results than the other embeddings in different scenarios: category classification, semantic similarity, and scientific concepts.

We have aimed at making FoodKG one of the best tools for data scientists and researchers in the FEW domains to develop next-generation applications using the concept of knowledge graphs and machine learning techniques. The rest of the paper is organized such that section 2 discusses recent related work; section 3 presents the design details of FoodKG; section 4 discusses the implementation and performance evaluation of FoodKG; and section 5 provides our conclusion.

## 2. Literature Review

FoodKG is a unique software in terms of type and purpose. There are no other systems or tools that have the same features. Our main work falls under graph embedding techniques. Embedded vectors learn the distributional semantics of words and are used in different applications such as Named Entity Recognition (NER), question answering, document classification, information retrieval, and other machine learning applications (Nadeau and Sekine, 2007). The embedded vectors mainly rely on calculating the angle between pairs of words to check the semantic similarity and perform other word analogy tasks, such as the common example *king - queen = man - woman*. The two main methods for learning word vectors are matrix factorization methods, such as Latent Semantic Analysis (LSA) (Deerwester et al., 1990), and Local Context Window (LCW) methods, such as skip-gram (Word2vec) (Mikolov et al., 2013b). Matrix factorization is the method that generates the low-dimensional word representation in order to capture the statistical information about a corpus by decomposing large matrices after utilizing low-rank approximations. In LSA, each row corresponds to a word or a concept, whereas columns correspond to a different document in the corpus. However, while methods like LSA leverage statistical information, they do relatively poor in the word analogy task, indicating a sub-optimal vector space structure. The second method aids in making predictions within a local context window, such as the Continuous Bag-of-Words (CBOW) model (Mikolov et al., 2013a). CBOW architecture relies on predicting the focus word from the context words. Skip-gram is the method of predicting all the context words one by one from a single given focus word. Few techniques have been proposed, such as hierarchical softmax, to optimize such predictions by building a binary tree of all the words then predict the path to a specific node. Recently, Pennington et al. (2014) shed light on GloVe, which is an unsupervised learning algorithm for generating embeddings by aggregating global word–word co-occurrence matrix counts where it tabulates the number of times word *j* appears in the context of the word *i*. FastText is another embedding model created by the Facebook AI Research (FAIR) group for efficient learning of word representations and sentence classification (Bojanowski et al., 2017). FastText considers each word as a combination of *n*-grams of characters where *n* could range from 1 to the length of the word. Therefore, fastText has some advantages over Word2vec and GloVe, such as finding a vector representation for the rare words that may not appear in Word2vec and GloVe. *n*-gram embeddings tend to perform better on smaller datasets.

A knowledge graph embedding is a type of embedding in which the input is a knowledge graph that leverages the use of relations between the vertices. We consider Holographic Embeddings of Knowledge Graphs (HolE) to be the state-of-art knowledge graph embedding model (Nickel et al., 2016). When the input dataset is a graph instead of a text corpus we apply different embedding algorithms, such as LINE (Tang et al., 2015), Node2ec (Grover and Leskovec, 2016), M-NMF (Wang et al., 2017), and DANMF (Ye et al., 2018). DeepWalk is one of the common models for graph embedding (Perozzi et al., 2014). DeepWalk leverages modeling and deep learning for learning latent representations of vertices in a graph by analyzing and applying random walks. Random walk in a graph is equivalent to predicting a word in a sentence. In graphs, however, the sequence of nodes that frequently appear together are considered to be the sentence within a specific window size. This technique also uses skip-gram to minimize the negative log-likelihood for the observed neighborhood samples. GEMSEC is another graph embedding algorithm that learns nodes clustering while computing the embeddings, whereas the other models do not utilize clustering. It relies on sequence-based embedding with clustering to cluster the embedded nodes simultaneously. The algorithm places the nodes in abstract feature space to minimize the negative log-likelihood of the preserved neighborhood nodes with clustering the nodes into a specific number of clusters. Graph embeddings hold the semantics between the concepts in a better way than word embeddings, and that is the reason behind using a graph embedding model to utilize graph semantics in FoodKG.

## 3. Methodology

We presented a domain-specific tool, FoodKG, that solves the problem of repeated, unused, and missing concepts in knowledge graphs and enriches the existing knowledge by adding semantically related domain-specific entities, relations, images, and semantic similarities values between the entities. We utilized AGROVEC, a graph embedding model to calculate the semantic similarity between two entities, get most similar entities, and classify entities under a set of predefined classes. AGROVEC adds the semantic similarity scores by calculating the cosine similarity for the given vectors. The triple that holds the semantic score will be encoded as a blank node where the subject is the hash of the original triple; the relation will remain the same, and the object is the actual semantic score.

**Figure 1:**
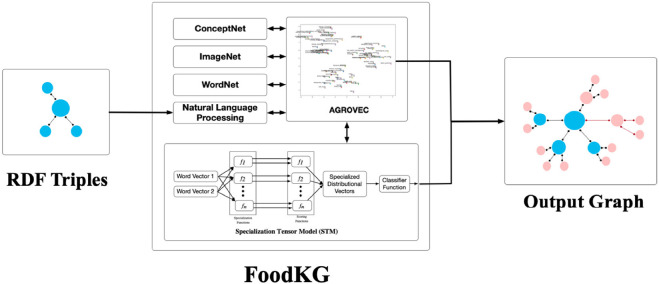
FoodKG system architecture.

FoodKG will parse and process all the subjects and objects within the provided knowledge graph. For each subject, a request will be made to WordNet to fetch its offset number. WordNet is a lexical database for the English language where it groups its words into sets of synonyms called synsets with their corresponding IDs (offsets). FoodKG requires these offset numbers to obtain the related images from ImageNet since the existing images on ImageNet are organized and classified based on the WordNet offsets. ImageNet is one of the largest image repositories on the Internet, and it contains images for almost all-known classes (Chen et al., 2018). These images will be added to the provided graph in the form of triples where the subject is the original word, the predicate will be represented by “#ImgURLs,” and the object is a Web link URL that contains the images returned from ImageNet. Figure 1 depicts the FoodKG system architecture.

### 3.1. AGROVEC

**Figure 2:**
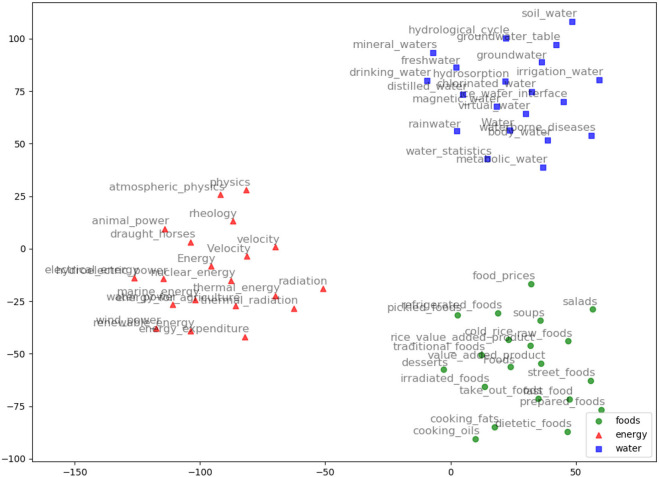
AGROVEC embeddings visualization using TSNE for the words: Food, Energy, and Water with their top 20 nearest neighbors based on AGROVEC model. The figure shows how AGROVEC cluster similar concepts together properly.

AGROVEC is a domain-specific embedding model that uses GEMSEC, a graph embedding algorithm. It was retrained and fine-tuned on AGROVOC, to produce a domain-specific embedding model. The embedding visualization (using TSNE; van der Maaten and Hinton, 2008) for our clustered embeddings is depicted in Figure 2. AGROVEC has the advantage of clustering compared to other models. ARGOVEC was trained with a 300-dimension vector and clustered the dataset into 10 clusters. The Gamma value used was 0.01. The number of random walks was six with windows size six. We started with the default initial learning rate of 0.001. AGROVEC was trained using the AGROVOC dataset that contains over 6 Million triples to construct the embedding.

### 3.2. Entity Extraction

FoodKG provides several features; entity extraction is one of the most important features. Users can start by uploading their graphs to FoodKG. Most of the provided graphs contain the same repeated concepts and terms that were named differently (e.g., id, ID, _id, id_num, etc.) where all of them represent the same entity, and other terms use abbreviations, numbers, or short-forms (acronyms) (Shen et al., 2015). Similar entities with different names create many repetitions and make it a challenge for different graphs to merge, search, and ingest in machine learning or linked data. To overcome this issue, we ran NLP techniques, such as POS tagging, chunking, and Stanford Parser, over all the provided subjects to extract the meaningful classes and terms that can be used in the next stage. For example, the following subjects “CHEESE,COTTAGE,S-,W/FRU” and “BUTTER,PDR,1.5OZ,PREP,W/1/1.HYD,” will be represented in FoodKG as “Cheese Cottage” and “Butter,” respectively. Users have the option of whether to provide the context of their graphs or leave it empty.

### 3.3. Text Classification

Nowadays, there are many different models to classify a given text to a set of tags or classes, such as the Long Short Term Memory (LSTM) network (Sachan et al., 2019). Nevertheless, text classification is still a challenge when it comes to classifying a single word without a context: “apple” has a broad context, for example, and the word could refer to many different things other than apple the fruit. Therefore, few solutions have been proposed, such as referring to fruits with small letters “apple” and capital letters for brand names like “Apple” the corporation. However, such a technique does not seem to be working well in large-scale contexts. Besides, this technique does not work in all domains since domain-specific graphs may not include all different contexts for a given word. Therefore, for each domain-specific area, there should be a knowledge graph that researchers and scientists can use in their experiments. At this point, our tool, FoodKG, becomes helpful to build and enrich such knowledge graphs and classify words to a specific class. FoodKG uses AGROVEC to help in providing the context for such scenarios. We use a simple yet effective technique with the help of ConceptNet API to accomplish this task (Speer et al., 2017). The idea is to start with a set of predefined classes; for example, let us consider only two classes for now, such as “fruits” and “animals.” After running these classes on ConceptNet, we store all returned top related concepts with the relation “type_of.” Here is an example of the returned words for “fruit”: *pineapple, mango, grapes, plums, berry, etc*.. The returned words for “animals” are *lion, fish, dolphin, fox, pet, deer, etc*.. We get only the top 10 instances from each category to limit the time complexity of our algorithm. Then, using AGROVEC embeddings, we calculate the semantic similarity score between the given word and all the other words from each class and return the highest average between them (Algorithm 1). Based on the highest average score, we choose the category of the given word. This technique proved to be the most reliable technique when it comes to classifying a category using word embeddings. The algorithm time complexity is O(N), where N is the number of classes that we started with. As an example, AGROVEC predicted the class “Food” for the concept “brown_rice,” “Energy” class for the concept “radiation,” and “Water” for the concept “hail.”

**Algorithm 1:** Text Classification using AGROVEC and ConceptNet

Input: *Target, Cat* = {*A*_1_ = {*word*_1_, …, *word*_10_}, …, *A*_*N*_ = {*word*_1_, …, *word*_10_}}
Output: The predicted class for the *Target*

1: function Loop(*Cat*[], *Target*)
2: *prediction* ← *nil*
3: *highestAvg* ← 0
4: *N* ← *length*(*Cat*)
5: for *i*←1 to *N* **do**
6: *total, Avg* ← 0
7: *K* ← *length*({*A*_*i*_})
8: for *j*←1 to *K* **do**
9: *total* ← *total* + *cosineSimilarity*(*Target, A*_*i*_[*j*])
10: end **for**
11: *Avg* ← *total*/*K*
12: if *Avg* > *highestAvg* **then**
13: *highestAvg* ← *Avg*
14: *prediction* ← *A*_*i*_
15: end **if**
16: end **for**
17: return *prediction*
18: end **function**

### 3.4. Semantic Similarity

Measurement of the semantic similarity between two terms or concepts has gained much interest due to its importance in different applications, such as intelligent graphs, knowledge retrieval systems, and similarity between Web documents (Iosif and Potamianos, 2010). Several semantic similarity measures have been developed and used based on this purpose (Martinez-Gil, 2014). In this paper, we adopted the cosine similarity measurement to measure the similarity between two vectors. FoodKG uses the semantic similarity measure between different subjects in a given graph. The semantic similarity scores will be attached as blank nodes to the original triple where the subject is the hashed blank node ID, the relation is “#semantic_similarity,” and the object the similarity score. These similarity scores can be used in different recommendation systems, question answering, or in future NLP models. FoodKG relies on the AGROVEC embedding model to generate the similarity scores. Table 1 shows the semantic similarity scores generated by AGROVEC and other models. This example was taken from the AGROVOC dataset to show how the AGROVEC model ranks these pairs in a better way than the other models. Table 2 shows the top five related words for “Food,” Table 3 shows the top five related words for “Energy,” and Table 4 shows the top five related words for “Water.”

**Table 1:** An example on how each model ranks the objects when the subject is “wheat” AGROVEC ranks the semantic similarity scores accurately from closest to furthest from the subject.

**Object**	**AGROVEC**	**HolE**	**GloVe**	**Word2vec**	**fastText**
Wheat_flour	0.757	−0.199	0.295	0.948	0.992
Barley	0.523	0.868	0.421	0.741	0.976
Grapes	0.116	0.851	0.802	0.930	0.885
Tuna_oil	0.046	−0.769	0.376	0.524	0.940
Building_components	0.016	0.923	0.397	0.466	0.883

**Table 2:** Top 5 related words for the concept “Foods.”

**Model**	**Top 5 related words**
AGROVEC	traditional_foods, soups, raw_foods, value_added_product, cooking_fats
HolE	controls, sterilizing, consumer_expenditure, Andean_Group, structural_crops
GloVe	meat, animal_meals, milk, water, seaweeds
Word2vec	cocoa_beans, hides_and_skins, eggs, oilseed_protein, soyfoods
fastText	pet_foods, raw_foods, seafoods, soyfoods, skin_producing_animals

**Table 3:** Top 5 related words for the concept “Energy.”

**Model**	**Top 5 related words**
AGROVEC	nuclear_energy,energy_for_agriculture,energy_expenditure, animal_power,renewable_energy
HolE	stored_products_pests, plant_breeding, age, formulations, sewage
GloVe	Ericales,carbohydrates,Sphingidae,Orobanchaceae,fungal_spores
Word2vec	stray_voltage_effects, irrigation_canals, libraries, agencies, CMS bioenergy,computer_science,wood_energy,cytogenetics
fastText	Cytogenetics

**Table 4:** Top 5 related words for the concept “Water.”

**Model**	**Top 5 related words**
AGROVEC	hydrosorption, chlorinated_water, water_statistics, body_water, virtual_water
HolE	isObjectOfActivity,dissolved_oxygen,economic_competition, state,international_cooperation
GloVe	seaweeds, meat, perishable_products, phosphorus, drugs
Word2vec	quarters, meat_byproducts, captivity, magnetic_water, plant_parts
fastText	heaters, bound_water, low_water, esters, high_water

### 3.5. Scientific Terms

Researchers and data experts often use domain-specific terms and concepts that may not be commonly used. For instance, these terms *Triticum, Malus, and Fragaria* are the scientific names for *wheat, apples, and strawberries*, respectively. However, such names may not exist in global word or knowledge graph embedding models. As for FEW, these terms can be found in our embedding model since it was trained on AGROVOC terms. This allows data experts to use similar scientific names and other related terms under the food domain while using FoodKG. Table 5 shows an example of top related concepts for FEW that do not exist in global embeddings.

**Table 5:** Few examples for the most used concepts in FEW domain that do not appear in global embeddings.

**Food**	**Energy**	**Water**
cocoa_products	energy_balance	water_activity
brown_rice	energy_generation	water_extraction
gluten_free_bread	energy_consumption	water_availability
skim_milk	energy_value	water_quality
emmental_cheese	energy_resources	water_statistics

### 3.6. Relationship Prediction

Word embeddings are well-known in the world of NLP due to their powerful way of capturing the relatedness between different concepts. However, capturing the lexico-semantic relationship between two words (i.e., the predicate of a triple) is a critical challenge for many NLP applications and models. Few techniques have been developed previously that proposed modifying the original word embeddings to include specific relations while training the corpora (Faruqui et al., 2015; Mrkšić et al., 2017; Vulić and Mrkšić, 2018). These approaches have used the post-processing trained embeddings to check the concepts that move closer together or further apart toward a specific relation. While these algorithms were able to predict specific relations like synonyms and antonyms, predicting, and discriminating between multiple relations is still a challenge. To overcome this challenge, we used transfer learning using the state-of-art STM model, that outperforms previous state-of-art models on CogALex and WordNet datasets, and the AGROVOC dataset to predict domain-specific relations between two concepts. The newly derived model aims particularly at classifying relations between different subjects in the food, agriculture, energy, and water domains.

## 4. Evaluation

In this section, we report the evaluation of AGROVEC and compare it with other word and knowledge graph embedding techniques: GloVe, fastText, Word2vec, and HolE.

### 4.1. Evaluation Technique

We employed the Spearman rank correlation coefficients (Spearman's rho; Myers and Sirois, 2004) in order to evaluate the embedding models. Spearman's rho is a non-parametric measure for assessing the similarity score between two variables. We applied Spearman's rho between the predicted cosine similarity using the embeddings and the ground truth, which is known as the relatedness task (Schnabel et al., 2015). When the ranks are unique, the Spearman correlation coefficient can be computed using the formula:

(1) $$\begin{array}{l} R_{s}=1-\frac{6\overset{n}{\underset{i=1}{\sum }}D_{i}^{2}}{n\left(n^{2}-1\right)} \end{array}$$

where *D*_*i*_ is difference between the two ranks of each observation and *n* is the total number of observations.

**Listing 1:**
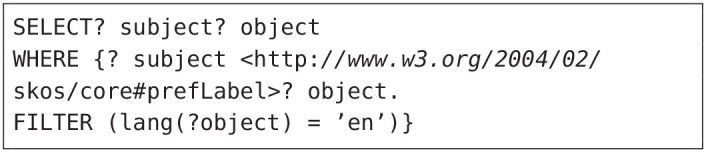
SPARQL query to extract the English triples

### 4.2. Dataset Description

AGROVOC is a collection of vocabularies that covers all areas of interest to the Food and Agriculture Organization of the United Nations, including food, nutrition, agriculture, fisheries, forestry, and the environment. It comprises of 32,000 concepts, in over 20 languages, where each concept is represented using a unique id. For instance, the subject “http://aims.fao.org/aos/agrovoc/c_12332” corresponds to “maize.” We used the SPARQL query in listing 1 to extract English triples.

### 4.3. Benchmark Description

While there exist well-known word-embedding benchmark datasets, such as WordSim-353 (Finkelstein et al., 2002), for evaluating the semantic similarity measures, these cannot be employed for domain-specific embeddings since many concepts related to FEW are not considered in public benchmarks. Constructing a domain-specific benchmark is a challenge considering the need for domain experts. Therefore, we leverage ConceptNet to construct a benchmark dataset for evaluating the models. ConceptNet originated from the crowd-sourcing project Open Mind Common Sense, which was launched in 1999 at the MIT Media Lab. ConceptNet used to be a home-grown crowd-sourced project with the purpose of improving the state of computational knowledge and human knowledge. However, currently, the data is generated from many trusted resources such as WordNet, DBPedia, Wiktionary (Zesch et al., 2008), OpenCyc (Smywiński-Pohl, 2012), and others. We split AGROVOC dataset based on its 126 unique relations to depict how each model performs against the different relations and to study the impact of the number of hops between the concepts in the embedding. For each subject and object, we looked up the weights returned from ConceptNet and considered them to be the ground truth.

### 4.4. Results

We evaluated two recent graph embedding models, namely DeepWalk and GEMSEC, trained on AGROVOC data, to analyze their performance on the FEW domains. Table 6 reports the average Spearman correlation coefficient scores for DeepWalk and GEMSEC. The higher score attained by GEMSEC motivated us to use GEMSEC for constructing AGROVEC.

**Table 6:** Different graph embedding techniques with their Spearman Correlation score.

**Model description**	**Spearman Correlation**
DeepWalk (Perozzi et al., 2014)	0.068
GEMSEC (Rozemberczki et al., 2019)	0.101

**Figure 3:**
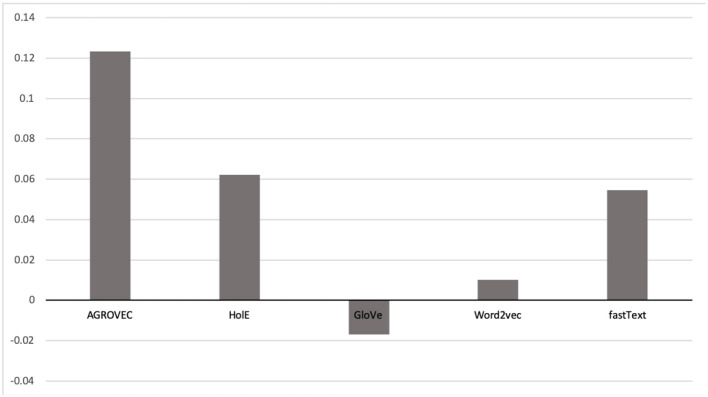
Spearman correlation coefficient ranking scores compared against ConceptNet. This figure shows how AGROVEC scored highest scores, which means its ranking is the closest for ConceptNet ranking (all models trained on AGROVOC dataset and tested against the same benchmark).

**Figure 4:**
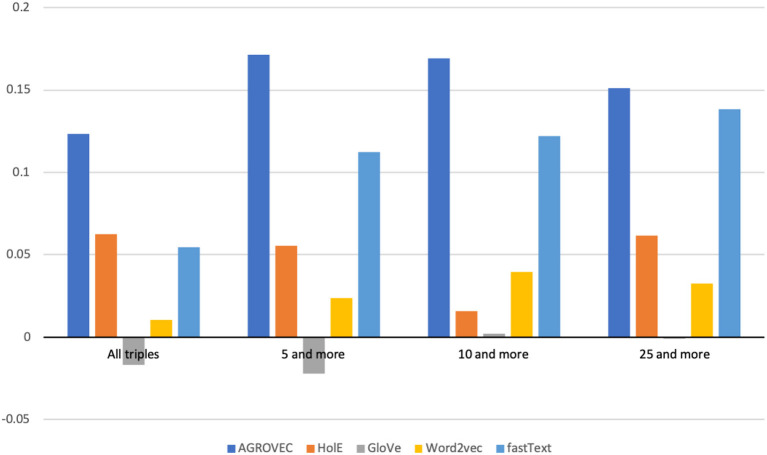
Spearman correlation coefficient scores when evaluated on all triples and relations with minimum number of word pairs in each relation being 5, 10, and 25.

**Figure 5:**
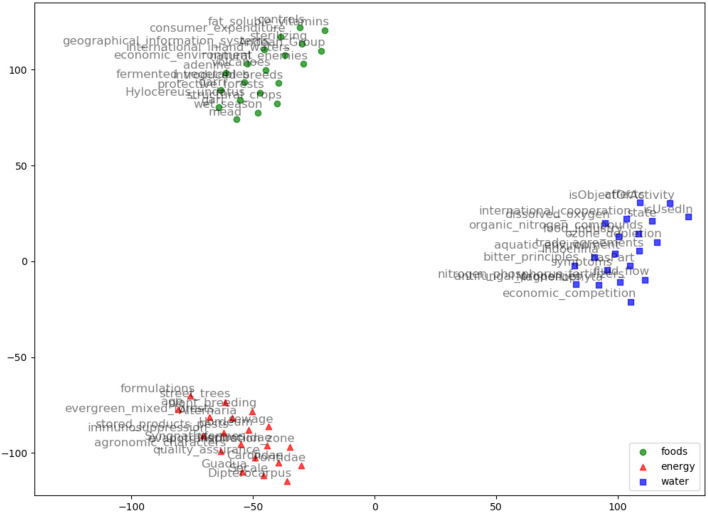
HolE embeddings visualization using TSNE for the words: Food, Energy, and Water with their top 20 nearest neighbors based on HolE model.

**Figure 6:**
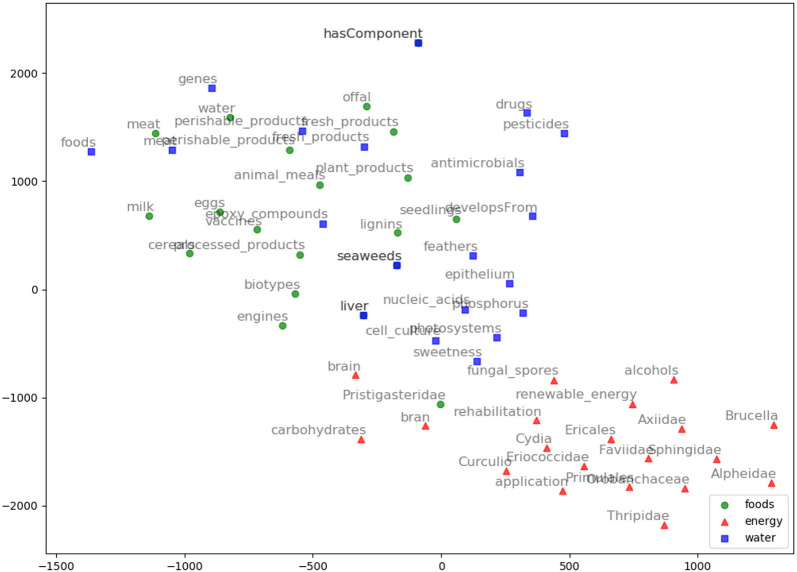
GloVe embeddings visualization using TSNE for the words: Food, Energy, and Water with their top 20 nearest neighbors based on GloVe model.

**Figure 7:**
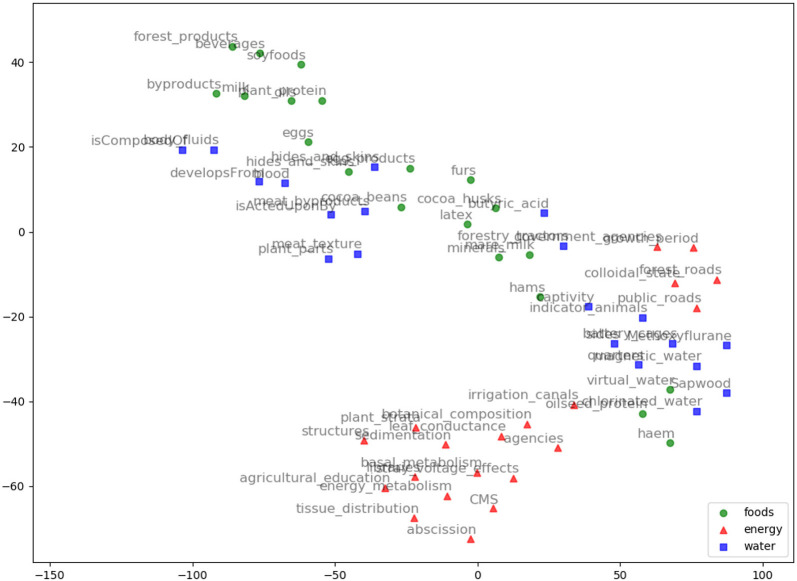
Word2vec embeddings visualization using TSNE for the words: Food, Energy, and Water with their top 20 nearest neighbors based on Word2vec model.

**Figure 8:**
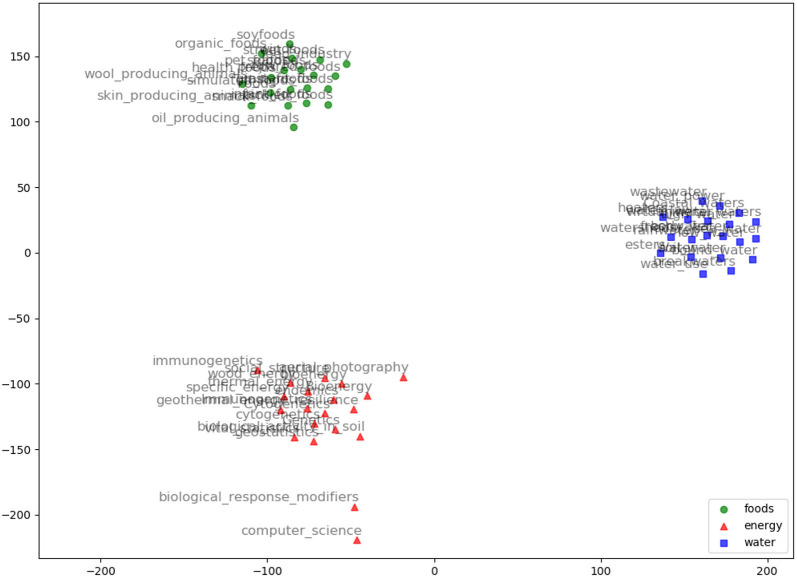
FastText embeddings visualization using TSNE for the words: Food, Energy, and Water with their top 20 nearest neighbors based on fastText model.

We evaluated AGROVEC against HolE, GloVe, Word2vec, and fastText, where all of the models were retrained using their default parameters on the AGROVOC dataset except for the number of dimensions. The number of dimensions used for all models was 300, with the minimum count set as 1 to include all the concepts and relations. Figure 3 shows the average Spearman correlation coefficient scores for all the models evaluated on 126 unique relations. Figure 4 shows the Spearman correlation coefficient scores while limiting the minimum number of word pairs in each relation to 5, 10, and 25 in order to check the model's performance across the different number of word pairs. The results show that AGROVEC, based on GEMSEC trained and fine-tuned on AGROVOC, outperforms all other models by a significant margin when predicting FEW domain similarity scores. Figure 2 shows an example of the AGROVEC embedding using TSNE for the domains Food, Energy, and Water with the top 20 related terms. This shows how these domains with their top related terms are properly clustered. However, Figures 5–8 visualize how Hole, GloVe, Word2vec, and fastText cluster Food, Energy, and Water domains with their top 20 related terms. From Figures 2, 5–8 we observe that AGROVEC achieves better clustering, with the terms of the same domain being placed closer. This was because AGROVEC uses GEMSEC which uses self clustering.

We also compared the top five related terms for food, energy, and water, as detailed in Tables 2–4, respectively. While AGROVEC, which uses GEMSEC trained and fine-tuned on AGROVOC, was able to fetch appropriate concepts related to the provided terms, the other models struggled despite being trained on the same dataset using their default parameters without fine-tuning.

## 5. Conclusion

In this paper, we presented FoodKG, a novel software tool to enrich knowledge graphs constructed on FEW datasets by adding semantically related knowledge, semantic similarity scores, and images using advanced machine learning techniques. FoodKG relies on AGROVEC, which was constructed using GEMSEC but retrained and fine-tuned on the AGROVOC dataset. Since AGROVEC was trained on a controlled vocabulary, it provides more accurate results than global vectors in the food and agriculture domains for category classification and semantic similarity of scientific concepts. The STM model, retrained on the AGROVOC dataset, is used for the prediction of semantic relations between graph entities and classes. The output produced by FoodKG can be queried using a SPARQL engine through a friendly user interface. We evaluated AGROVEC using the Spearman Correlation Coefficient algorithm, and the results show that our model outperforms the other models trained on the same graph dataset.

## Data Availability Statement

The datasets analyzed for this study can be found in the [AIMS (AGROVOC)] http://aims.fao.org/vest-registry/vocabularies/agrovoc. FoodKG code can be found at this Github repository https://github.com/Gharibim/FoodKG.

## Author Contributions

All authors have been involved in the design, development, and evaluation of the software. They have also been involved in writing different parts of the paper.

## Conflict of Interest

The authors declare that the research was conducted in the absence of any commercial or financial relationships that could be construed as a potential conflict of interest.
