# Corrigendum: FoodKG: A Tool to Enrich Knowledge Graphs Using Machine Learning Techniques

**DOI:** 10.3389/fdata.2020.00021

**Published:** 2020-06-10

**Authors:** Mohamed Gharibi, Arun Zachariah, Praveen Rao

**Affiliations:** ^1^Department of Computer Science and Electrical Engineering, University of Missouri-Kansas City, Kansas City, MO, United States; ^2^Department of Electrical Engineering and Computer Science, University of Missouri-Columbia, Columbia, MO, United States; ^3^Department of Health Management and Informatics, University of Missouri-Columbia, Columbia, MO, United States

**Keywords:** machine learning, graph embeddings, knowledge graphs, AGROVOC, semantic similarity

In the published article, there was an error in the affiliation **of the first author**. Instead of the “**University of Missouri-Columbia**,” the university name should be “**University of Missouri-Kansas City**.”

The authors apologize for this error and state that this does not change the scientific conclusions of the article in any way. The original article has been updated.

